# An Assessment of Student Pharmacists’ Knowledge of Electronic Cigarettes or Vapes—A Cross Sectional Study at One College of Pharmacy

**DOI:** 10.3390/pharmacy10050131

**Published:** 2022-10-11

**Authors:** Ibrahim Alfayoumi, Osama Aqel, David R. Axon

**Affiliations:** Department of Pharmacy Practice and Science, R. Ken Coit College of Pharmacy, The University of Arizona, Tucson, AZ 85721, USA

**Keywords:** electronic cigarettes, knowledge, student pharmacists

## Abstract

This study assessed the knowledge of e-cigarettes/vapes among a sample of student pharmacists. A 22-item cross-sectional electronic questionnaire was administered to all third- and fourth-year student pharmacists enrolled at one college of pharmacy in the United States (N = 256). Data were collected over six weeks in March/April 2022. One point was assigned for each correct knowledge item; points were then summed to create a total knowledge score for each person. Differences in the proportion of students who correctly answered each knowledge item were compared between year groups using a chi-square test, while differences between year groups for total knowledge score were compared using a two-sample *t*-test. The a priori alpha level was 0.05. Fifty students (third year = 30, fourth year = 20; female = 60%) completed the survey. Students’ e-cigarettes/vapes knowledge varied depending on the item. There was no statistically significant difference between third- and fourth-year students for total mean knowledge scores (third year = 12.5 ± 3.3, fourth year = 11.2 ± 3.1, *p* = 0.1780) or for each knowledge item, except for items 10 and 20. In conclusion, the findings from this survey of student pharmacists at one college of pharmacy in the United States indicate a need for more education around e-cigarettes/vapes for student pharmacists so that they are better able to counsel patients on their use.

## 1. Introduction

The use of electronic cigarettes or vapes, also known as e-cigarettes, electronic nicotine delivery systems (ENDS), or vaping devices, has increased over the past decade and is a timely and significant public health issue [1]. As of February 2020, the United States (US) Centers for Disease Control and Prevention (CDC) reported 2807 hospitalized cases and 68 deaths attributed to electronic cigarettes or vaping-associated lung injury [1]. There are many different types of e-cigarettes/vapes, with some resembling pipes, cigars, “tank” devices, and others, but they all function similarly [2]. The components of an e-cigarette/vape include a battery, heating element, and chamber to hold the liquid [2]. E-cigarettes/vapes convert liquid nicotine into an aerosol or mist for the user to inhale into their lungs. The liquid usually contains nicotine and other added flavors [2]. The use of e-cigarettes/vapes can lead to nicotine addiction among non-smokers, in particular youngsters, or lead to a re-initiation of nicotine dependence in former smokers or increased severity of nicotine dependence in dual users of conventional cigarettes and e-cigarettes/vapes [3].

E-cigarettes/vapes are advertised as a smoking cessation aid, and as being healthier, cheaper, and more socially acceptable than conventional cigarettes [4]. While the possible long-term health effects of e-cigarettes/vapes are not yet clear, serious lung diseases can develop in some people who use e-cigarettes/vapes [5]. The symptoms of serious lung diseases include cough, breathing difficulties, chest pain, nausea, vomiting, diarrhea, fatigue, fever, or weight loss [5]. As of 18 February 2020, a total of 2807 cases severe enough to require hospitalization from all US states have been reported to the CDC; 68 deaths have been confirmed in 29 states and the district of Columbia [5]. However, it is not yet clear if all these cases have the same cause [5]. There are thousands of different e-cigarettes/vapes products available, and an even larger number of different nicotine liquids that can be used in them [5]. E-cigarettes/vapes were introduced to the US market in 2007 and their sales have doubled every year in the US since 2008 [6]. This increasing trend might be due to promotional campaigns for e-cigarettes/vapes on multiple mainstream marketing channels, including television, print, radio, and the Internet [6]. Most existing literature has focused on the effectiveness of e-cigarettes/vapes [7]. However, there is little published research regarding how healthcare professionals perceive the current use, status, and effectiveness of e-cigarettes/vapes [7].

A 56-item cross sectional survey of medicine, pharmacy, nursing, public health, and allied health students at a US academic health center evaluated the use, knowledge, and perceptions of e-cigarettes/vapes among healthcare students [8]. The study found the highest estimate of e-cigarettes/vapes use to date among healthcare students, and the perceptions of using e-cigarettes/vapes as smoking cessation aids, a perceived reduction in harm compared to tobacco, and a preference for reduced e-cigarettes/vapes regulation were all significantly associated with using e-cigarettes/vapes [8]. A further 33-item cross sectional survey regarding e-cigarettes/vapes was used to assess student pharmacists’ tobacco use, insights regarding e-cigarettes/vapes, smoking cessation education, and how their perception of e-cigarettes/vapes differ from those of other healthcare students (nursing, public health, optometry, dental hygiene, and others) at another US institution [9]. The study found that student pharmacists were less likely than other healthcare students to recommend the use of e-cigarettes/vapes to aid patients in cessation of traditional cigarette use [9].

However, there remains a lack of contemporary information about student pharmacists’ knowledge of e-cigarettes/vapes [10]. This information is important to know as pharmacists are frontline healthcare professionals often involved in facilitating smoking cessation [11]. The purpose of this study was to assess the knowledge and perceptions of e-cigarettes/vapes among student pharmacists at one college of pharmacy in the US.

## 2. Methods

### 2.1. Study Design and Eligibility Criteria

All third- and fourth-year student pharmacists currently enrolled at one college of pharmacy in the US were eligible to participate in this cross-sectional survey (total N = 256; third year N = 136, fourth year N = 130). Students who did not complete the survey were excluded. All students had completed a course on self-care pharmacotherapeutics in which they would have had an opportunity to learn about smoking cessation, and some students may have had opportunities to learn about e-cigarettes/vapes through their introductory or advanced pharmacy practice experiences (IPPEs/APPEs) or employment. However, students were not presented with any specific information about e-cigarettes/vapes immediately prior to participating in this survey.

### 2.2. Questionnaire

A 22-item questionnaire was developed using Research Electronic Data Capture (REDCap). REDCap serves as a secure, online platform to collect research data [12,13]. Students were asked to answer 20 multiple-choice knowledge questions (shown in Table 1) taken from the Tobacco Prevention Toolkit developed by the Division of Adolescent Medicine at Stanford University [14]. These 20 knowledge items asked about different aspects of e-cigarettes/vapes, including nine questions about nicotine, its effect on health and different amounts of nicotine in e-cigarettes/vapes e-liquids, six questions about different e-cigarettes/vapes e-liquid flavors and chemicals it contains, three questions about using e-cigarettes/vapes and associated health risks, and two questions about marketing e-cigarettes/vapes. In addition, there were two descriptive items (gender and academic year) to describe the study participants. The questionnaire was developed based on the literature with revisions made in an iterative fashion until the instrument was considered to have an appropriate content validity by the research team [8,9].

### 2.3. Data Collection

An email inviting all eligible students to participate in the online survey was sent in March 2022. The email contained information about the study and who to contact with questions, along with a link to complete the survey. A reminder email was sent after two weeks, and a further reminder email was sent after another two weeks. Data collection stopped in April 2022. The start of the questionnaire contained an informed consent document that participants were required to acknowledge before proceeding with the questionnaire.

### 2.4. Data Analysis

Each multiple-choice knowledge question was scored as correct or incorrect, with one point assigned for a correct answer and no points for an incorrect answer. Total scores for knowledge items could therefore range between 0 and 20. Differences in the proportion of students who correctly answered each knowledge item were compared between year groups using a chi-square test, while differences between year groups for total knowledge score were compared using a two-sample *t*-test. Differences in gender between year groups were compared using a chi-square test. The a priori alpha level was 0.05. However, a Bonferroni correction was applied to account for multiple comparisons with the chi-square test (0.05/20 comparisons = 0.0025). Thus, a *p*-value of < 0.0025 indicates significance for the chi-square comparisons. Data were analyzed using SAS Studio (v9.4, Cary, NC, USA).

## 3. Results

From the eligible population of 256 student pharmacists, 50 students (third year = 30, fourth year = 20) completed the survey and were included in the analysis (19.5% response rate). The majority of respondents were female (60%).

Table 1 reports the respondents’ knowledge of e-cigarettes/vapes. There was no statistically significant difference between third- and fourth-year students for each knowledge item (*p* > 0.0025) except for items 10 (How many flavors of e-cigarettes are currently being sold?) and 20 (What do we not know about pod-based systems?). There was also no statistically significant difference between third- and fourth-year students for total knowledge scores (third year mean total knowledge score = 12.5 ± 3.3, fourth year mean total knowledge score = 11.2 ± 3.1, *p* = 0.1780).

## 4. Discussion

The key findings from this study were that student pharmacists had a total mean knowledge score of 11.85 ± 3.2 out of 20, and that there were no statistically significant differences in knowledge of e-cigarettes/vapes between third- and fourth-year student pharmacists at one college of pharmacy in the US for total mean knowledge scores or for each knowledge item, except for items 10 and 20. These findings, and their implications for pharmacy education, are discussed below.

The finding that student pharmacists in this study had a total mean knowledge score of 11.85 ± 3.2 out of 20 indicates that students have some knowledge of e-cigarettes/vapes, although there are some gaps in their knowledge. There are limited studies of students in the US to compare our findings. However, a study in China showed that students’ level of knowledge regarding e-cigarettes/vapes was generally low [15]. For instance, approximately two-thirds of students were aware of the addictiveness of e-cigarettes, 51% of participants considered e-cigarettes to be carcinogenic, and fewer than one-third of students (31.1%) identified e-cigarettes as tobacco products [15]. To identify the specific areas where students’ knowledge of e-cigarettes/vapes was poor, we assessed each item individually.

Most student pharmacists in this study correctly answered items about nicotine (e.g., cravings, side effects, and the organs it affects). For instance, 90% of respondents correctly answered the item about a drop in nicotine levels causing nicotine cravings. These items were not necessarily specific to e-cigarette/vape products; thus, students may have been able to utilize their existing knowledge to answer these items. These findings suggest that students are likely receiving adequate education on nicotine through their didactic coursework, IPPEs/APPEs, and work experience. Likewise, most student pharmacists in this study correctly answered items about the marketing of e-cigarette/vape products. This is interesting given that a previous study conducted in Egypt among healthcare professionals and the general population found that media advertisements were the main source of information about electronic cigarettes [16]. These findings suggest that the marketing of e-cigarette/vape products may be influential in people’s knowledge regarding the perceptions of these products.

There was variation (depending on the specific item asked) in student pharmacists’ knowledge of the amount of nicotine and other chemicals in e-cigarette/vape products, ranging from as few as 16% of respondents correctly answering an item about the strength of nicotine in e-cigarettes/vapes devices to 58% of respondents correctly answering an item about the other chemicals in e-cigarettes/vapes. Furthermore, only 28% knew how many flavors of e-cigarettes/vapes were currently being sold, and 34% knew how e-cigarettes/vapes products deliver nicotine and other additives to the lungs. In particular, our study found that 48% of student pharmacists surveyed knew that e-cigarette/vape products sometimes contain nicotine. This finding correlates with those of another study conducted in Hangzhou, China, which found that 43% of student pharmacists thought e-cigarettes/vapes sometimes contain nicotine [15]. Another study found nursing students in the Philippines had poor knowledge (mean score 3.50 ± 1.64 out of 10) of e-cigarettes/vapes, particularly on the characteristics of e-cigarettes, chemical content, health effects, regulation status, and policies [17]. These findings provide evidence that various populations of students around the world have poor knowledge of e-cigarettes/vapes, which needs to be addressed.

These findings suggest that student pharmacists require further education specifically about e-cigarettes/vapes products to improve their knowledge and better prepare them to counsel patients. This is supported by the findings of a cross-sectional study of student pharmacists at one college of pharmacy in the US that found student pharmacists perceived themselves to be less knowledgeable about the harmful effects of e-cigarettes, the pharmacists’ role in counseling on e-cigarette cessation, and how patients can benefit from e-cigarette cessation counseling [18]. The study also found a higher proportion of students reported having no training on e-cigarette cessation compared to cigarette smoking cessation [18]. A similar study found that student pharmacists perceived themselves to be more knowledgeable about cigarette smoking cessation than about hookah tobacco use cessation, with 42.0% of respondents reporting that they thought hookah tobacco was less harmful than traditional cigarettes [19]. A further study assessed the content and extent of tobacco dependence education and intervention skills in US and Canadian dental schools and found that only 49% of faculty members surveyed were confident teaching students how to help patients cease tobacco use, and suggested that some faculty members lacked the interest and skills needed to teach tobacco cessation as part of patient care [20]. Targeted training on how to counsel patients on smoking cessation, including e-cigarettes and hookah cessation, should therefore be included in pharmacy curricula.

Our study also found that there were no differences in knowledge of e-cigarettes/vapes between the third- and fourth-year student pharmacists surveyed for total mean knowledge scores or for each knowledge item, except for items 10 and 20. This finding suggests that, in general, fourth-year students are not gaining additional knowledge of e-cigarettes/vapes during their APPEs or additional work experience opportunities, which may be another opportunity for students to learn about these products.

The limitations of this study include the nature of self-reported data from a survey with a relatively small sample size of 50 student pharmacists at one college of pharmacy in the US and the assumptions that students understood the survey questions and answered them accurately; thus, it lacks external validity beyond the population studied. Students had not received any information about e-cigarettes/vapes immediately prior to completing the survey; thus, they may not have had an accurate understanding of these products. The survey did not include a “don’t know” response option, so it may be possible that some students correctly guessed the answers to items that they did not actually know. Additional studies may be warranted to assess knowledge of e-cigarettes/vapes among a broader sample of student pharmacists and among other healthcare students to determine if the findings are similar. An educational intervention could also be designed to determine its usefulness for helping improve students’ knowledge of e-cigarettes/vapes.

## 5. Conclusions

This survey found that student pharmacists at one college of pharmacy in the US had a total mean knowledge score about e-cigarettes/vapes of 11.85 ± 3.2 out of 20, and that there were no statistically significant differences in knowledge of e-cigarettes/vapes between third- and fourth-year student pharmacists for total mean knowledge scores or for each knowledge item, for except items 10 and 20. These findings indicated that although some aspects of students’ knowledge were good, there were also some gaps in their knowledge of e-cigarettes/vapes. These findings suggest there is a need for student pharmacists to receive further education on e-cigarettes/vapes. To improve the generalizability of these results, further work is necessary to establish if these findings are similar among larger samples of student pharmacists and student pharmacists in other schools.

## Figures and Tables

**Table 1:** Student pharmacists’ knowledge of e-cigarettes.

Knowledge Questions	Third-Year Students (N = 30) N (%) Correct	Fourth-Year Students (N = 20) N (%) Correct	Total (N = 50) N (%)	*p*
1. E-cigarettes/vapes _____ have nicotine. Never ***Sometimes*** Always None of these	13 (43.3)	11 (55)	24 (48)	0.0980
2. Nicotine... Changes brain chemistry Is a stimulant Is highly addictive ***All the above***	24 (80)	15 (75)	39 (78)	0.3972
3. A drop in nicotine levels causes the body to have strong cravings for nicotine. ***True*** False	27 (90)	18 (90)	45 (90)	1.0000
4. Nicotine causes increased heart rate, lung damage, acid reflux, inhibits your sex drive and... Inhibits night vision ***More health problems for those with diabetes*** Hair growth None of these	24 (80)	16 (80)	40 (80)	1.0000
5. E-cigarette/vape pen flavors are fruit based and therefore not harmful. True ***False***	21 (70)	14 (70)	35 (70)	1.0000
6. E-cigarettes/vapes cause aerosols to enter the lungs, may contain nicotine, and causes ear/eye/throat irritation ***Yes*** No	27 (90)	15 (75)	42 (84)	0.0052
7. We know there are no health risks associated with the use of e-cigarettes/vape pens. True ***False***	22 (73.3)	12 (60)	34 (68)	0.0461
8. E-cigarettes are devices that deliver nicotine and/or additives in the form of a… Vapor ***Aerosol*** Steam Liquid	11 (36.7)	6 (30)	17 (34)	0.3150
9. E-cigarettes are also referred to as... Vape pens Pod-based Mods ***All the above***	22 (73.3)	12 (60)	34 (68)	0.0461
10. How many flavors of e-cigarettes are currently being sold? 12 1000 500 ***15,000+***	11 (36.7)	3 (15)	14 (28)	0.0005
11. Which of the following chemicals have been found in e-cigarettes/vape pens? Formaldehyde Arsenic Lead ***All the above***	19 (63.3)	10 (50)	29 (58)	0.0688
12. The amount of nicotine in an e-cigarette pod is nearly equivalent to: (pod is the piece that contains e-juice). 1 cigarette ***1.5 to 2 packs of cigarettes*** Half a pack of cigarette 5 packs of cigarettes	17 (56.7)	11 (55)	28 (56)	0.8087
13. Which of the following statements about e-cigarette’s liquids are TRUE? ***Some contain nicotine*** All contain nicotine None contain nicotine There are no flavors	14 (46.66)	9 (45)	23 (46)	0.8094
14. An e-cigarette/vape product with a 5% strength of nicotine is _________. Very low in nicotine Low in nicotine High in nicotine ***Very high in nicotine***	6 (20)	2 (20)	8 (16)	1.0000
15. Which organs in the human body does nicotine affect? Stomach Heart Lungs ***All the above***	24 (80)	15 (75)	39 (78)	0.3972
16. Vaping labs reports 7 ingredients in their pod e-juice. Independent scientists found how many chemicals in their pod e-juice? 7 ***59*** 19 They have not studied that yet	10 (33.3)	8 (40)	18 (36)	0.3255
17. What about pod-based system advertisements makes it problematic for young people? The young-looking model targets young people The use of specific words stands out to young people The pod-based company uses social justice imagery ***All the above***	24 (80)	15 (75)	39 (78)	0.3972
18. How are pod-based systems marketed to young people? Use of flavors and colors Misleading labeling of nicotine Advertisements including people that look like young people ***All the above***	26 (86.7)	15 (75)	41 (82)	0.0355
19. Market e-juices range from 0–25 mg per ml of nicotine, while one pod has at least ___ mg of nicotine per pod. 4.13 mg/mL 5 mg/mL 25 mg/mL ***41.3 mg/mL***	6 (16.7)	3 (15)	9 (18)	0.7420
20. What do we NOT know about pod-based systems? All the specific ingredients Long-term effects of using it Effects of nicotine on the brain ***All the specific ingredients and long-term effects of using it***	27 (90)	14 (70)	41 (82)	0.0004

The correct answer to each item is indicated in italics and bold below the item.

## Data Availability

Data are available from the corresponding author upon reasonable request.
